# Circulating tumour DNA analysis in multiple myeloma

**DOI:** 10.18632/oncotarget.21595

**Published:** 2017-10-07

**Authors:** Sridurga Mithraprabhu, Andrew Spencer

**Affiliations:** Andrew Spencer: Australian Centre for Blood Diseases, Monash University, Malignant Haematology & Stem Cell Transplantation, Alfred Hospital and Department of Clinical Haematology, Monash University, Melbourne, Australia

**Keywords:** multiple myeloma, cell-free DNA, circulating tumour DNA, mutations, therapeutic monitoring

For over a decade the possibility of cancer diagnosis and characterisation through a blood test rather than repeated, invasive, and often uninformative tissue biopsies has been explored. This is now feasible via the interrogation of circulating free tumour derived DNA (ctDNA)—short fragments of DNA shed from tumours into the plasma that harbor mutations present in both primary tumours and metastases [1]. Such analysis is now frequently integrated into clinical trials and plasma DNA EGFR mutation testing for non-small cell lung cancer has recently been approved by the FDA [2]. Further commercialisation of these ‘liquid biopsies’ as diagnostics is rapidly evolving but currently is largely limited to improving treatment choices in late stage cancers.

Multiple myeloma (MM) is a malignancy of terminally differentiated plasma cells that is largely confined to the intra-medullary bone marrow (BM) milieu. MM is a markedly heterogeneous multi-focal disease that manifests complex cytogenetic and molecular abnormalities including primary translocations involving the immunoglobin heavy chain locus and driver and/or secondary mutations involving numerous oncogenic signalling pathways. The conventional approach for monitoring MM tumour burden is via quantitation of serum biomarkers - clonal immunoglobulin (paraprotein) and/or isotype restricted free-light chains (serum free light chains). However, these approaches are inadequate to define minimal residual status (MRD) and uninformative in subsets of patients with oligo-secretory (OS) or non-secretory (NS)-MM. The genomic characterisation of the disease, most frequently at diagnosis, is achieved via sequential testing of single-site BM biopsies, a strategy that clearly fails to accommodate the perceived clonal heterogeneity and multi-focal nature (spatial heterogeneity) of the disease. As such there remains a critical need for newer strategies that will enable both comprehensive mutational characterization in MM and a more practical approach to evaluating treatment response in the more challenging subsets of MM. Recently published data would suggest that ctDNA analysis may represent such an approach.

The evaluation of ctDNA for mutational characterisation and monitoring of disease burden in MM has recently been described [3-6] with the levels of cell-free DNA being significantly higher in patients with MM compared to normal volunteers and non-MM cancers [3, 6]. Importantly, and for the first time, spatial and clonal heterogeneity in MM was confirmed by our study, with a high sensitivity targeted sequencing platform demonstrating that 21% of MM patients had mutations detectable only in the plasma and not BM [3]. While it is almost certain, with high-sensitivity approaches, that there will be a mutant allele fraction (MAF) threshold for minor BM sub-clones that enables them to be reproducibly detected in the plasma this is likely not relevant to less sensitive strategies that cannot detect smaller sub-clonal mutations, consistent with the 96% concordance between BM and PL demonstrated using next-generation sequencing (NGS) technologies [6]. However, confirmatory studies are required to validate these observations.

BM whole exome sequencing (WES) studies in MM have demonstrated activating mutations of the RAS-MAPK pathway in approximately 50% of patients [7, 8]. In contrast, our study demonstrated mutations in 69% of cases and the co-existence of multiple mutated sub-clones in a significant proportion, with >3 mutations in 23% of patients (range, 3-17 mutations per patient) representing a hitherto unrecognized mutational convergence on the RAS-MAPK pathway. This has remained largely undiscovered with single-site BM WES studies likely owing to the relative insensitivity of the methodologies utilised and the presence of undetected clonal heterogeneity at sites distant to the BM biopsy sites. Another interesting and novel observation is the presence of predominantly plasma-based *PIK3CA* mutations as described in the paper from Kis et al (2017). These have rarely been described previously in MM and the detection in the plasma raises the possibility that they may be a feature of extramedullary (EM) disease. Therefore, plasma ctDNA analysis for EM patients may provide insight into the aetiology of EM disease and could theoretically also furnish information on response to therapy through sequential tracking of plasma-only mutations.

Oberle and colleagues assessed clonotypic V(D)J rearrangement in cell-free DNA in a cohort of 27 MM patients and an association between the presence of cfDNA V(D)J rearrangements with response to therapy was demonstrated [4]. Similarly, Rustaad *et al* and our group have tracked ctDNA quantifiable somatic mutations suggesting the capacity with such an approach to predict disease response and relapse [3, 5]. However, all of these studies were limited by small sample sizes and a lack of homogenous treatment and monitoring strategies. Moreover, a significant shortcoming of assessing a specific mutant clone over several years is the possibility of emergent sub-clonal mutations associated with disease relapse. We therefore propose that sequential assessment, preferably with a targeted high-sensitivity platform, capable of quantitating existing and new sub-clones in the plasma, is necessary to comprehensively monitor patients. The accumulative published experiences and imminent developments in the field of ctDNA analysis indicate that this type of analysis will, in the near future, provide critical information for precision medicine and likely transform the management of problematic sub-groups of MM including both NS and OS patients and those with EM disease.

## References

[1] Siravegna G (2017). Nat Rev Clin Oncol.

[2] Cobas EGFR Mutation Test v2. 2016. http://www.fda.gov/Drugs/InformationOnDrugs/ApprovedDrugs/ucm504540.htm

[3] Mithraprabhu S (2017). Leukemia.

[4] Oberle A (2017). Haematologica.

[5] Rustad EH (2017). Haematologica.

[6] Kis O (2017). Nat Commun.

[7] Lohr JG (2014). Cancer Cell.

[8] Walker BA (2015). J Clin Oncol.

